# Programming Positive Mechanofluorescence in Liquid Crystalline Elastomers

**DOI:** 10.1021/acsapm.3c01050

**Published:** 2023-08-02

**Authors:** Pedro Güixens-Gallardo, Ignacio Brea, Jordi Manrique, Farhad Shohraty, Jaume Garcia-Amorós, Dolores Velasco

**Affiliations:** † Grup de Materials Orgànics, Departament de Química Inorgànica i Orgànica (Secció de Química Orgànica), Universitat de Barcelona, Martí i Franquès 1, E-08028 Barcelona, Spain; ‡ Institut de Nanociència i Nanotecnologia (IN2UB), Universitat de Barcelona, E-08028 Barcelona, Spain

**Keywords:** elastomers, liquid crystals, luminescence, fluorescent molecular rotors, positive mechanofluorescence, sensors, smart materials

## Abstract

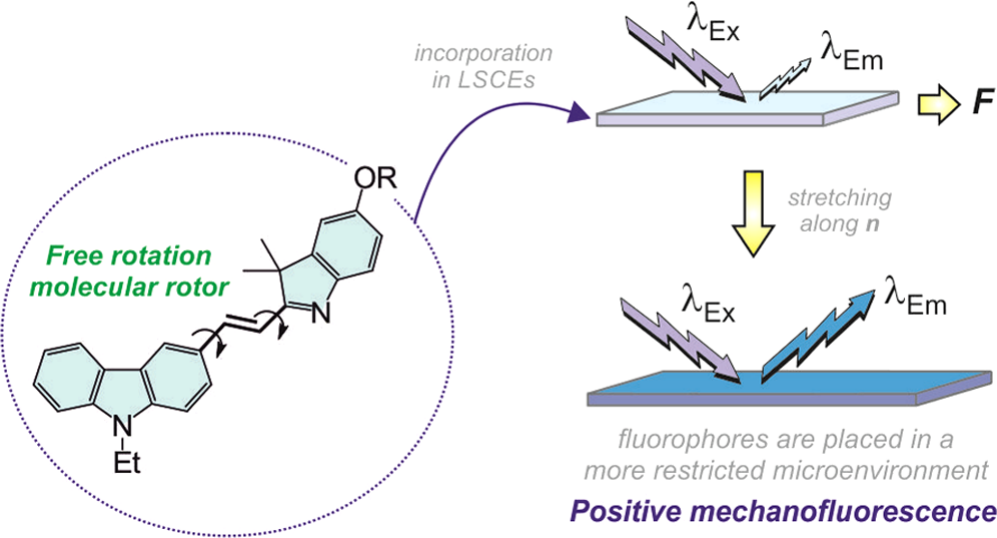

Liquid single crystal elastomers (LSCEs) containing organic fluorophores within their polymeric network are attractive materials to detect forces with simple spectroscopic measurements. Hitherto, all mechanoluminescent LSCEs decrease their emission intensity upon mechanical stimulation; that is, they display negative mechanofluorescence. Such behavior is governed by the mechanically induced approximation of the quenching mesogenic units and the fluorophores. In this work, we propose the integration of fluorescent molecular rotors (FMRs), whose luminescence is not quenched by the mesogens, in LSCEs as a valuable strategy to conceive elastomeric materials programmed with exactly the opposite behavior, i.e., their fluorescence increases upon deformation (positive mechanofluorescence). Specifically, carbazole-indolenine dyes are interesting candidates for this purpose since their luminescence depends mainly on the degree of intramolecular rotation allowed by the local environment. On this basis, the uniaxial deformation of an LSCE, along its anisotropic direction, incorporating such FMRs will place the fluorophores in a more restricted medium, leading to the desired enhanced emission at the macroscale.

## Introduction

Carbazole is a recurrent and highly versatile scaffold within chemistry, biomedicine, and materials science because of its attractive luminescent and semiconductor properties, which can be fine-tuned through not only the subtle and rational modification of its molecular structure but also imparting control over the interactions established with the environment at the supramolecular level.^1−12^

Liquid crystalline elastomers (LCEs) are macromolecular materials showing a unique combination of a self-organized nature and entropic elasticity.^13,14^ Such confluence has enabled the development of smart functional materials by means of the covalent attachment of stimuli-responsive moieties (like azobenzene derivatives) to their polymeric network.^15−20^ In particular, our research group has been engaged during recent years in the design and study of force-sensing materials by introducing carbazole fluorophores into LSCEs (LCEs displaying a common orientation of the directors). These materials exhibit a quick and noticeable decrease in their fluorescent emission when they are stretched along their anisotropic direction. The initial state of the system is rapidly restored once the external force is removed. In this way, the application of mechanical forces to LSCEs furnishes two distinguishable states (*ON* and *OFF*) with distinct emission. Thanks to this phenomenon, carbazole-containing LSCEs can be exploited as optical mechanotransducers, i.e., materials capable of detecting mechanical stimuli and converting them into processable optical signals.^21^ As a whole, materials that modulate their emission of light upon the application of forces and other external stimuli have sparked a great interest recently because of their widespread applications.^22−40^

The mechanofluorescence of our materials arises from the increment in the nematic order that occurs when they are stretched along the director direction. During the uniaxial deformation of the sample, both mesogen and carbazole molecules are brought closer in space, and a more favorable overlap between each other is induced. The enhanced interaction between the quenching mesogens and the carbazole fluorophores results in a suppression of their fluorescence, which translates into an overall decrease of the emission intensity of the whole material (negative mechanofluorescence).

In previous studies, we have shed light on the structural factors that govern the intermolecular interactions established between the mesogenic and the fluorogenic units, which, in turn, determine the mechanofluorescent response observed macroscopically.^41,42^ Specifically, we have explored how the anchoring mode (side-on vs end-on) of the carbazole fluorophores to the elastomeric network influences the sensing abilities of the final material.^43^ Similarly, we have studied the luminescent response when applying mechanical forces as a function of the length of the flexible alkyl chain connecting the fluorophore to the main polymer backbone.^44^ However, all of the optical mechanotransducers based on LSCEs reported so far decrease their emission intensity upon deformation.

**Figure 1:**
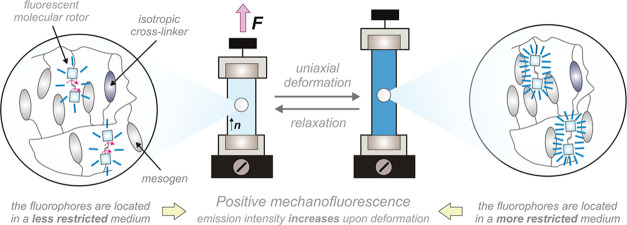
Schematic illustration of the phenomenon of positive mechanofluorescence.

In this work, we target the development of a novel class of liquid crystalline elastomeric materials exhibiting the exact opposite behavior; that is, their fluorescent emission increases as a response to an external mechanical stimulation (positive mechanofluorescence, Figure 1). This behavior has never been programmed in LSCEs before. For this purpose, a complete redesign of the intermolecular interactions established between the mesogens and the fluorophores at the nanoscale has to be envisioned in order to unlock the desired response at the macroscopic level. Nevertheless, it should be stressed at this point that conventional non-liquid crystalline elastomeric materials showing an increase of their fluorescent emission upon the application of mechanical forces have been widely studied and successfully applied for different purposes like, for instance, molecular damage sensing.^45^ Furthermore, fluorescence activation as a result of mechanochemical reactions has also been largely exploited in diverse applications.^46,47^

Herein, we propose the integration of fluorescent molecular rotors (FMRs) in LSCEs as a valuable strategy to engineer optical mechanotransducers with positive mechanofluorescence. FMRs are fluorescent molecules whose emission is significantly dependent on the degree of intramolecular rotation permitted by the medium. Briefly, in those media where rotational freedom is not hindered, the fluorophores dissipate their excitation energy through a nonradiative pathway; radiative deexcitation predominates when the local environment becomes restricted (like the one induced in the LSCE after its deformation).^48−51^ Our approach consists of the implementation of FMRs into an elastomeric matrix that does not absorb in the emission range of the fluorophore. In this way, the stretching of the LSCE will not result in a quenching of the emission of the fluorophores by the mesogens but will place the former in a more restricted microenvironment, thereby giving rise to the sought-after enhancement of the material fluorescence.

In this paper, we devise for the first time the design schemes required to program positive mechanofluorescence in nematic LSCEs. Furthermore, a comprehensive study of the luminescent properties of the FMRs used will allow us to rationalize this effect and open the possibility of further developing LSCE-based force sensors with unprecedented abilities and improved performances.

## Results and Discussion

### Design and Synthesis

**Figure 2:**
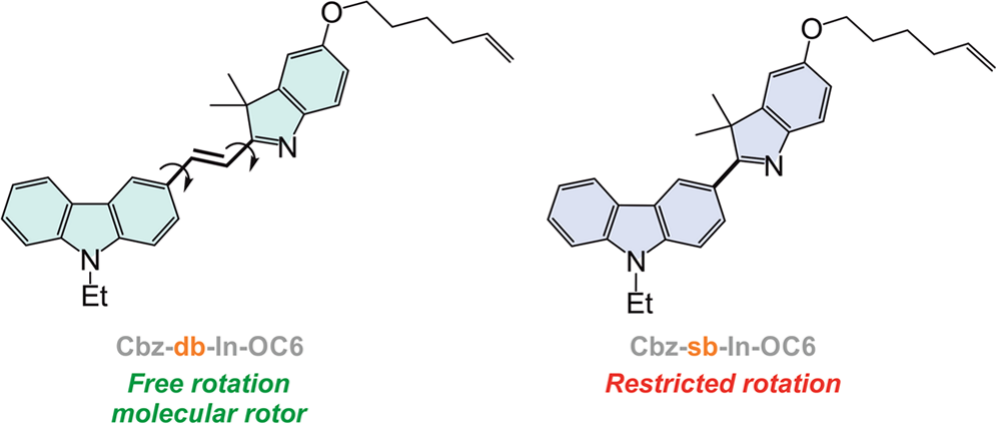
Chemical structure of the fluorophores proposed to program positive mechanofluorescence in LSCEs.

For the present study, two different fluorophores have been considered, which combine a carbazole and an indolenine platform within their molecular structure (**Cbz-sb-In-C6** and **Cbz-db-In-C6** in Figure 2). The main difference between both fluorophores is that the former displays the two heterocycles connected directly by a single C–C bond (sb in Figure 2) while the latter has them linked through a double C=C bond (db in Figure 2). Importantly, this structural feature allows the double-bonded fluorophore to exhibit molecular rotor-like behavior. In fact, FMRs are π-conjugated molecules whose fluorescence intensity varies with the degree of intramolecular rotation of the rotators incorporated in the π-conjugated bridge, which is significantly influenced by the free volume of the microenvironment where they are located.^49^ In addition, both molecules incorporate a peripheral 5-hexenyl chain, placed at position 5 of the indolenine ring, to allow their covalent attachment to the elastomeric network.

**Figure 3:**
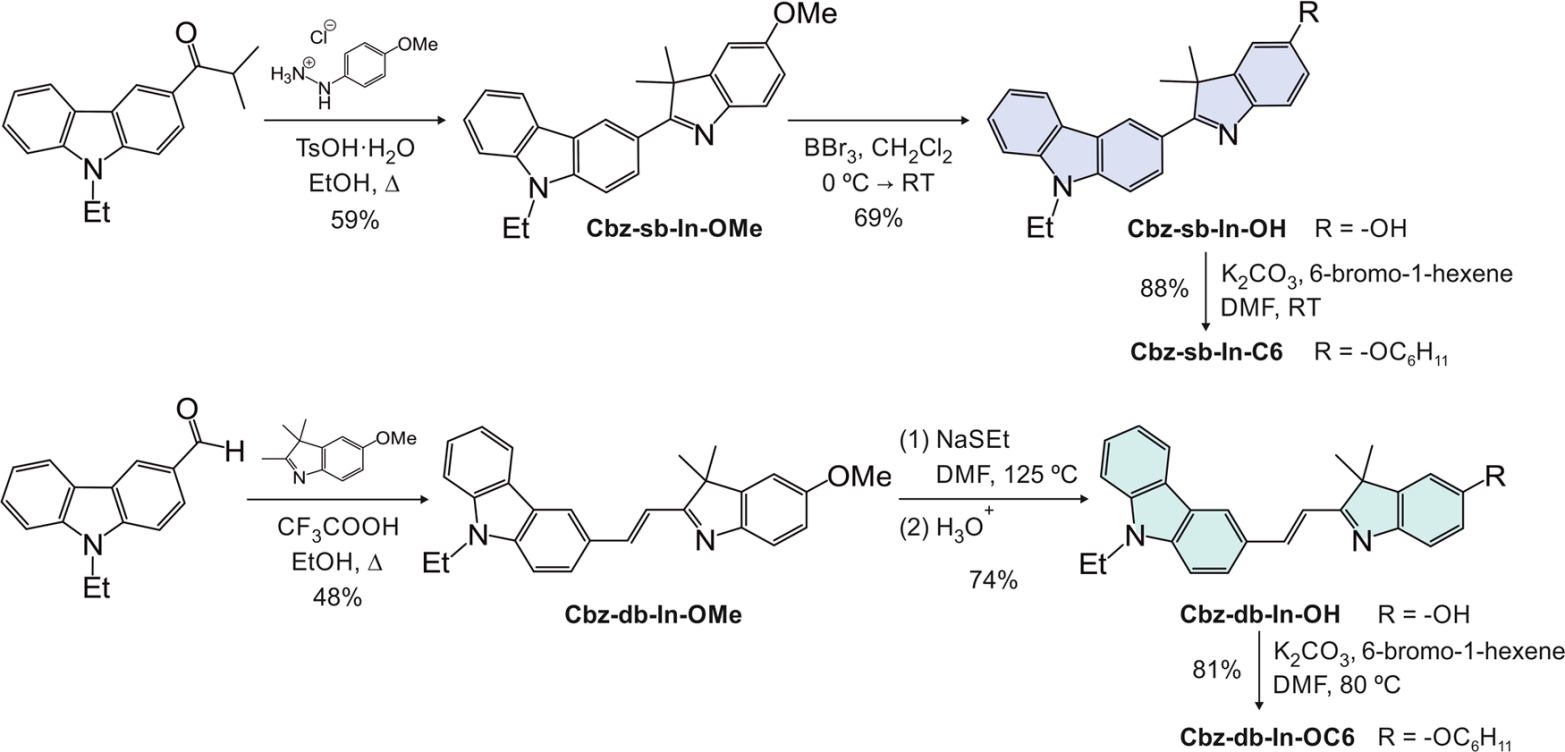
Synthetic route for the fluorophores **Cbz-sb-In-C6** and **Cbz-db-In-C6**.

1-(9-Ethyl-9*H*-carbazol-3-yl)-2-methylpropan-1-one and 5-methoxy-2,3,3-trimethyl-3*H*-indole were synthesized following literature procedures.^52,53^ The preparation of **Cbz-sb-In-C6** (Figure 3) starts with a Fischer indole synthesis between 4-methoxyphenyl-hydrazine hydrochloride and 1-(9-ethyl-9*H*-carbazol-3-yl)-2-methylpropan-1-one in the presence of *p*-toluenesulfonic acid monohydrate in boiling ethanol. Next, the methyl ether of **Cbz-sb-In-OMe** was cleaved with boron tribromide in dichloromethane at room temperature, thereby obtaining the corresponding phenolic derivative **Cbz-sb-In-OH**. In the last synthetic step, the OH group was alkylated via a Williamson reaction using potassium carbonate as a base and 6-bromo-1-hexene as the alkylating agent in DMF at room temperature. Fluorophore **Cbz-sb-In-C6** was obtained in an overall yield of 36%. The synthesis of **Cbz-db-In-C6** (Figure 3) initiates with a Knoevenagel condensation between 9-ethyl-9*H*-carbazole-3-carbaldehyde and 5-methoxy-2,3,3-trimethyl-3*H*-indole in the presence of trifluoroacetic acid in boiling ethanol. In this instance, the methyl ether of **Cbz-db-In-OMe** was cleaved with sodium ethanothiolate in DMF at 125 °C. Finally, the alkylation of **Cbz-db-In-OH** with K_2_CO_3_ and 6-bromo-1-hexene at 80 °C yielded the target fluorophore. **Cbz-db-In-C6** was obtained in an overall yield of 29%. The identities of all compounds were confirmed by ^1^H NMR spectroscopy and high-resolution mass spectrometry. Detailed synthetic protocols and structural data concerning the final fluorophores and the corresponding intermediates are reported in the Supporting Information.

### Absorption and Emission Spectroscopies

**Figure 4:**
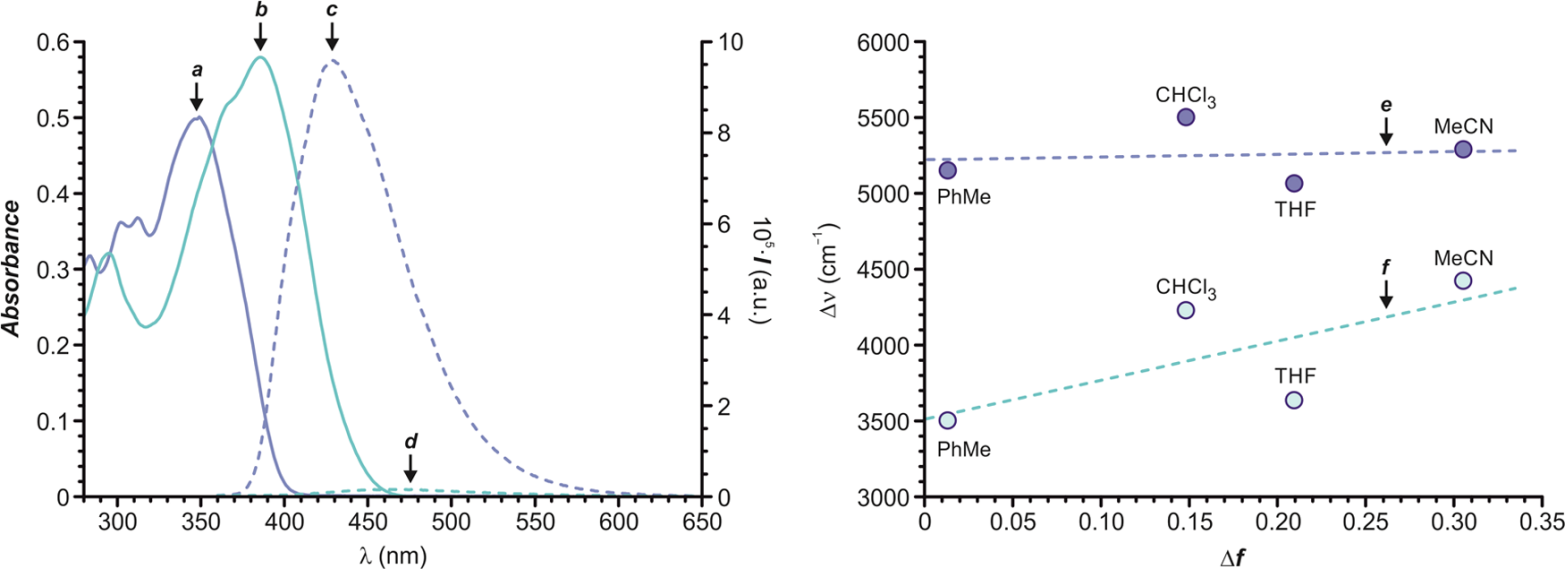
Absorption (a, b) and emission (c, d, λ_Ex_ = 340 nm) spectra of acetonitrile solutions (20 μM) of **Cbz-sb-In-OMe** (a, c) and **Cbz-db-In-OMe** (b, d). Lippert–Mataga plot for **Cbz-sb-In-OMe** (e) and **Cbz-db-In-OMe** (f).

The simplest monomeric units that allow us the spectroscopic study in solution of the two fluorophores described previously are represented by **Cbz-sb-In-OMe** and **Cbz-db-In-OMe**. Compound **Cbz-sb-In-OMe** absorbs entirely within the UV region of the electromagnetic spectrum (a in Figure 4), exhibiting its wavelength of maximum absorption (λ_Abs_ in Table 1) at 347–351 nm. The introduction of the π-bridge allows conjugation between the carbazole and indolenine units and leads to a significant bathochromic shift of *ca*. 40 nm. Accordingly, **Cbz-db-In-OMe** absorbs within the UV-blue zone (b in Figure 4), with a λ_Abs_ of 385–390 nm. Both compounds emit mainly blue light when excited within their absorption band. Compound **Cbz-sb-In-OMe** shows its wavelength of maximum emission (λ_Em_ in Table 1) at 421–435 nm, while **Cbz-db-In-OMe** displays a red-shifted λ_Em_ at 449–467 nm (c, d in Figure 4, respectively), as a consequence of the extended electronic conjugation of the two heterocyclic moieties.

**Table 1:** Wavelength of Maximum Absorption (λ_Abs_), Molar Extinction Coefficient (ε), Wavelength of Maximum Emission (λ_Em_), Stokes Shift (Δν), and Fluorescence Quantum Yield (Φ_F_) for Compounds Cbz-sb-In-OMe and Cbz-db-In-OMe in Different Solvents

	solvent	λ_Abs_ [nm]	ε [mM^–1^ cm^–1^]	λ_Em_ [nm]	Δν [cm^–1^]	Φ_F_
Cbz-sb-In-OMe	toluene	348	23.8	424	5151	0.24
	chloroform	351	21.9	435	5502	0.51
	tetrahydrofuran	347	24.0	421	5065	0.31
	acetonitrile	349	25.1	428	5289	0.22
Cbz-db-In-OMe	toluene	388	25.3	449	3501	0.012
	chloroform	390	25.9	467	4228	0.019
	tetrahydrofuran	386	29.8	449	3635	0.011
	acetonitrile	385	29.0	464	4422	0.005

The Stokes shift of the fluorophores (Δν in Table 1) was calculated in various solvents to identify possible solvatochromic effects. Indeed, the Lippert–Mataga plot is an intuitive way to detect solvatochromism by representing the Stokes shift of a compound versus its orientation polarizability (Δ*f* in Figure 4) in a specific solvent. It is well known that the carbazole heterocycle is a weak electron donor, and thus, it does not produce a noticeable intramolecular charge transfer within the molecule. As a consequence, the absorption and emission of **Cbz-sb-In-OMe** and **Cbz-db-In-OMe** are barely affected by solvent polarity as it is revealed by the corresponding Lippert–Mataga plots (e, f in Figure 4, respectively). Therefore, we conclude that both fluorophores lack solvatochromism. The absorption and emission spectra of **Cbz-sb-In-OMe** and **Cbz-db-In-OMe** in the different solvents analyzed are reported in Figure S2 in the Supporting Information.

**Cbz-db-In-OMe** is much less fluorescent than **Cbz-sb-In-OMe** independently of the polarity of the solvent used. In particular, **Cbz-sb-In-OMe** has a relative fluorescence quantum yield (Φ_F_ in Table 1) ranging from 0.22 to 0.51. On the other hand, **Cbz-db-In-OMe** has very poor Φ_F_ values around 0.010. The much lower fluorescence quantum yield detected for **Cbz-db-In-OMe** can be ascribed to the dynamic intramolecular rotation of the aryl rings along the C–C bonds, thereby losing its excitation energy following nonradiative pathways.^54^

### Viscosity Dependence of the Luminescence

**Figure 5:**
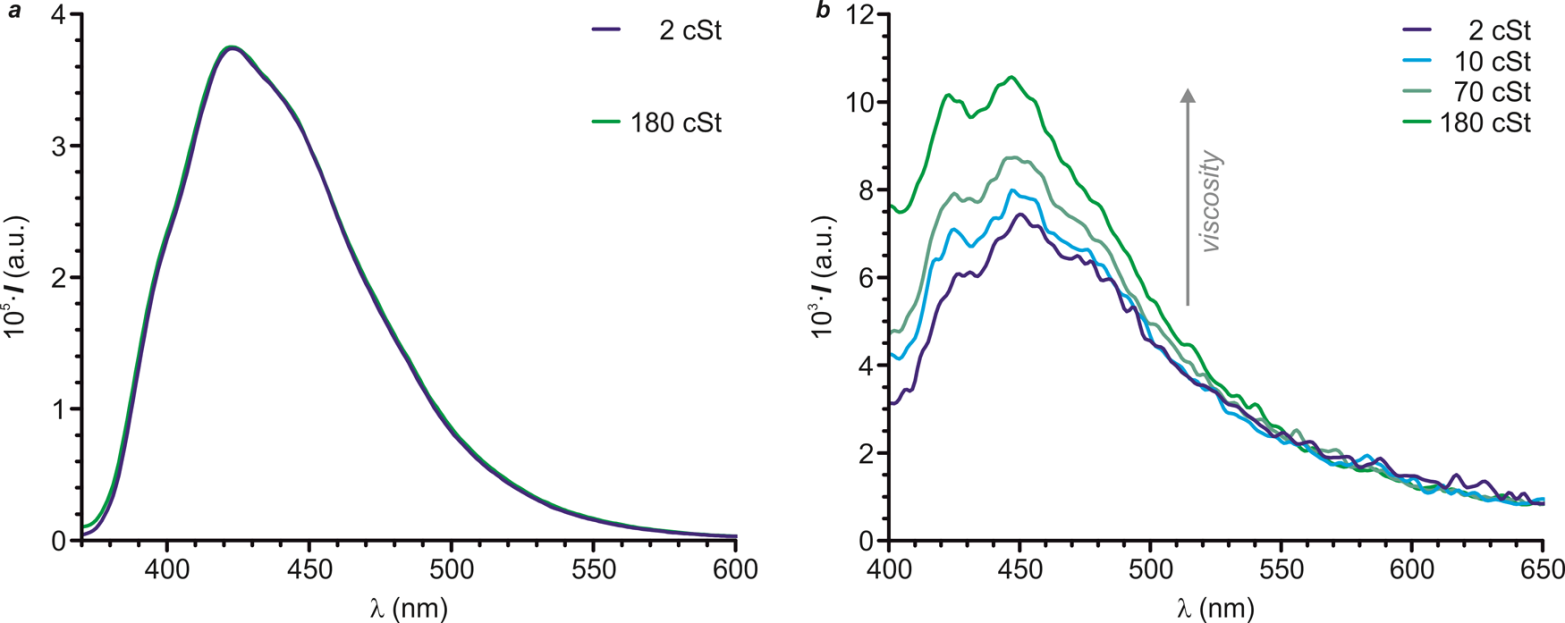
Emission spectra of 3 μM solutions of **Cbz-sb-In-OMe** (a) and **Cbz-db-In-OMe** (b) in toluene–PDMS mixtures of increasing viscosity.

The potential behavior of **Cbz-sb-In-OMe** and **Cbz-db-In-OMe** as FMRs has been tested by analyzing the viscosity dependence of their fluorescence emission in solution. In order to mimic as much as possible the host matrix and the intermolecular interactions established by the fluorogenic monomers with the local environment within the LSCE, we have used binary mixtures of toluene, an aromatic conventional solvent, and poly(dimethylsiloxane) (PDMS; molecular weight = 117,000 g·mol^–1^), a siloxane-based viscous polymer similar to the one used in the LSCEs (*vide infra*). The kinematic viscosity (ν) of the different toluene–PDMS solutions was measured with a Cannon–Fenske viscometer. The range of ν values of the toluene–PDMS mixtures spans from 2 to 180 cSt at room temperature. Importantly, the emission spectrum of **Cbz-sb-In-OMe** does not change upon increasing the viscosity of the medium (a in Figure 5). On the other hand, the emission intensity of **Cbz-db-In-OMe** increases gradually when measured in more viscous toluene–PDMS mixtures (b in Figure 5). This differential behavior evidences that **Cbz-db-In-OMe** operates indeed as an FMR and, therefore, it is a good candidate to be introduced in nematic LSCEs to achieve optical mechanotransducers with positive mechanofluorescence.

### Preparation and Characterization of the LSCEs

**Figure 6:**
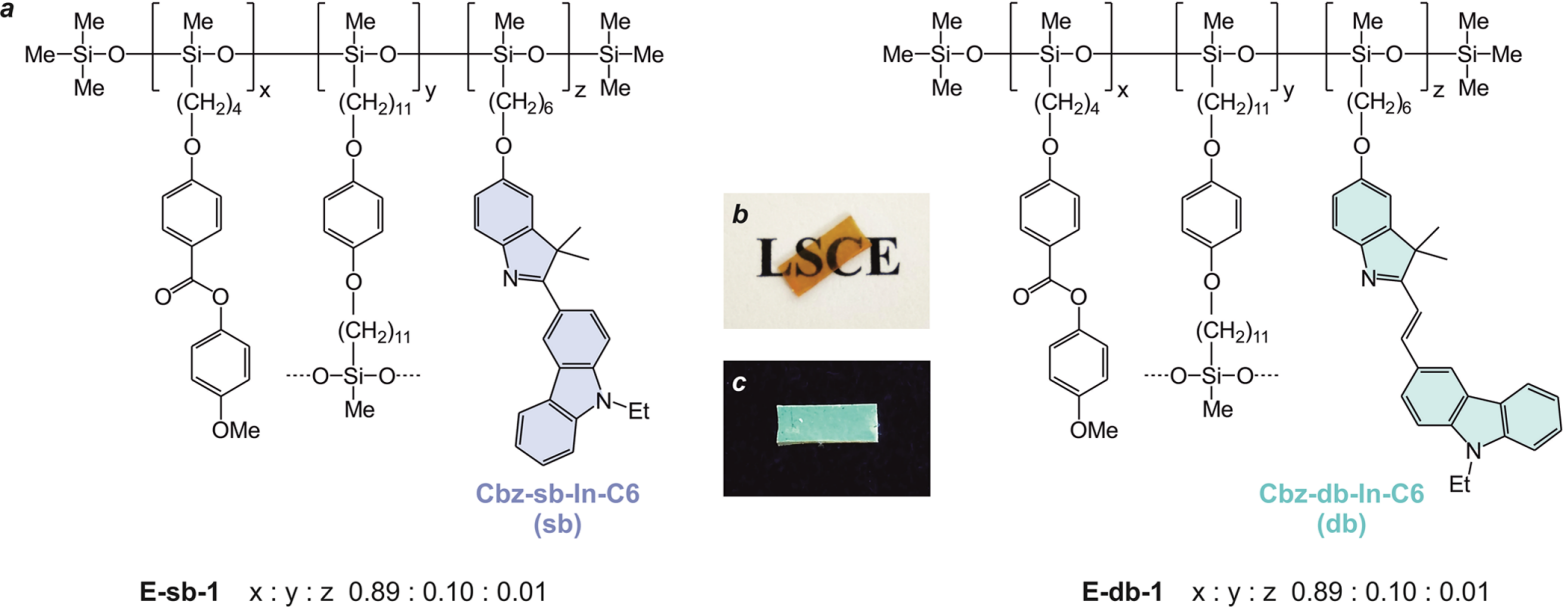
Chemical structure (a) of the LSCEs **E-sb-1** and **E-db-1**. Picture of the elastomer **E-db-1** under ambient light (b) and UV light (c, λ_Ex_ = 365 nm).

LSCEs have been synthesized following the procedure in three steps developed by Küpfer and Finkelmann over the 1990s.^55^Figure 6 shows the chemical structure of the different elastomers prepared. The elastomeric network is formed by a polyhydrogenomethylsiloxane backbone cross-linked by 1,4-di(10-undecenyloxy)benzene (10% mol, **CL** in Figure S1). The systems also contain 4-methoxyphenyl 4-(3-butenyloxy)benzoate (89% mol, **M4OMe** in Figure S1), the mesogen that provides the liquid crystalline nature, and either one of the fluorophores **Cbz-sb-In-C6** or **Cbz-db-In-C6** (1% mol), which emit light in the form of fluorescence. The different monomers were covalently attached to the main polymer backbone via a Pt-catalyzed hydrosilylation reaction. The protocol for the preparation of the LSCEs is detailed in the Supporting Information.

Two nematic LSCEs containing 1% mol of dye have been prepared successfully using either of the two fluorophores. It should be emphasized that the fluorophore content has been kept as low as possible (1) because the obtained fluorescent response produced by the LSCEs was intense enough to evaluate their mechanoluminescence and (2) in order not to disturb the self-organization of the host elastomeric matrix. The elastomers prepared are denoted as follows: E-*m*-*n*, where E means elastomer, *m* indicates the fluorophore incorporated into the LSCE (in this instance, *sb* and *db* will be used as a shorthand notation for **Cbz-sb-In-C6** and **Cbz-db-In-C6**, respectively), and *n* informs about its content in the elastomeric material.

The mesophase exhibited by the elastomers under ambient conditions was established by X-ray diffraction (XRD). The broad reflex found in the wide-angle region [2θ ∼ 19.7, spacing ∼4.5 Å (*d* in Table 2)] of the diffractogram (a, b in Figure S3 in the Supporting Information) indicates the existence of a nematic phase at 298 K. In addition, the highly anisotropic azimuthal intensity distribution of this reflex (c, d in Figure S3 in the Supporting Information), with intensity maxima at 90 and 270°, suggests that monodomain elastomeric materials have been obtained. Specifically, the order parameter (*S* in Table 2) of the mesogenic units has been estimated to be *ca*. 0.55 for both elastomers. Accordingly, polarized optical microscopy experiments manifest the expected change from darkness to brightness (Figure S4 in the Supporting Information) when the analyzer of the optical microscope is rotated by 45° with respect to the main axis of the sample, which is ascribable to the characteristic monodomain structure of LSCEs. The thermal range of the stability of the nematic phase has been assessed by differential scanning calorimetry (DSC). DSC thermograms (Figure S5 and S6 in the Supporting Information) reveal that **E-sb-1** and **E-db-1** adopt an enantiotropic nematic phase before isotropization. Indeed, the nematic-to-isotropic phase transition is observed at 332–339 and 343 K (*T*_N–I_ in Table 2), respectively, with an associated enthalpy (Δ*H*_N–I_ in Table 2) of about 1.8 J·g^–1^. Both systems exhibit a glass transition below room temperature. Thus, DSC analyses confirm that all LSCEs exhibit a nematic mesophase under the experimental conditions at which the mechanofluorescence of the LSCEs is evaluated. The effective cross-linking density of the elastomeric samples was analyzed by means of swelling experiments. The swelling ratio (*q* in Table 2), , where  and  are the ratios between the dimensions in the deswollen liquid crystalline state and in the swollen isotropic state in the different directions with respect to the nematic director (***n***), was calculated to be 3.1 and 3.6 for LSCEs **E-sb-1** and **E-db-1**, respectively.

**Table 2:** Distance between the Mesogenic Units (*d*), Nematic Order Parameter (*S*), Nematic-to-Isotropic Phase Transition Temperature (*T*_N–I_), Nematic-to-Isotropic Phase Transition Enthalpy (Δ*H*_N–I_), and Swelling Parameter (*q*) for the LSCEs E-sb-1 and E-db-1

			*T*_N–I_ [K]		Δ*H*_N–I_ [J·g^–1^]		
elastomer	*d* [Å]	*S*	cooling	heating	cooling	heating	*q*
**E-sb-1**	4.5	0.55	332	339	2.1	1.5	3.1
**E-db-1**	4.5	0.56	343	343	2.0	1.6	3.6

### Mechanofluorescence of the LSCEs

**Figure 7:**
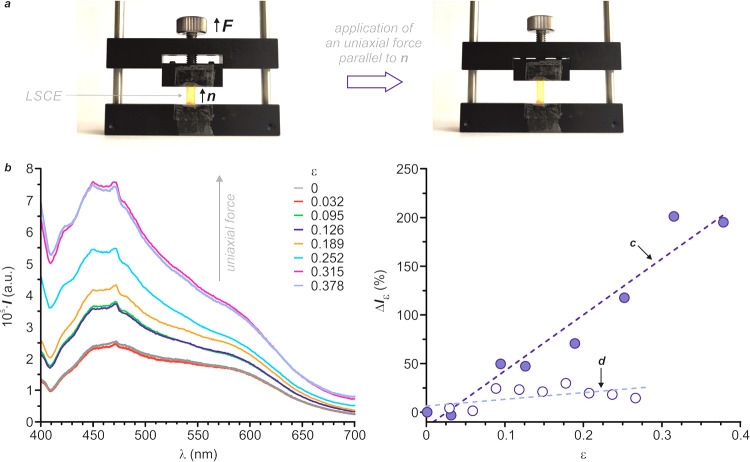
Mechanofluorescence of the LSCEs: (a) elongation of the nematic LSCE **E-db-1** by applying a uniaxial force along ***n***. Evolution of the emission spectrum for **E-db-1** (b) and of the relative emission intensity upon deformation, along the director direction, for LSCEs **E-db-1** (c, λ_Ex_ = 365 nm, λ_Em_ = 475 nm) and **E-sb-1** (d, λ_Ex_ = 365 nm, λ_Em_ = 438 nm).

Mechanofluorescence experiments involve monitoring the emission intensity (*I*) of the LSCE, while it is progressively deformed by means of the application of a mechanical force parallel to its longest axis, that is, along the anisotropic direction of the sample (a in Figure 7). In all cases, the fluorogenic moieties have been excited with UV light (λ_Ex_ = 365 nm for **E-sb-1** and **E-db-1**). For **E-sb-1**, *I* (or in relative terms, Δ*I*_ε_ = (*I*_ε_ – *I*_0_/*I*_0_) · 100, where *I*_ε_ and *I*_0_ are the fluorescence intensities of the stretched and the relaxed sample, respectively) at λ_Em_ = 438 nm suffered only a minimal variation (around 20%) after the stretching of the material (d in Figure 7). In contrast, **E-db-1** experiences a linear and significant increase in its emission intensity upon the application of the mechanical force (b, c in Figure 7). Specifically, this elastomer intensifies its fluorescent emission by *ca*. 200% at λ_Em_ = 475 nm under a deformation of ε = 0.32. Such an enhancement in the emission intensity before and after elongation generates two interconvertible states for the system and enables to turn on its fluorescence under mechanical control (positive mechanofluorescence). Remarkably, elastomer **E-db-1** is, to the best of our knowledge, the first LSCE-based force sensor reported in the literature that increases its fluorescence emission upon the application of a mechanical force.

The driving force of the mechanofluorescence displayed by our elastomeric materials is the increase in the nematic order of the LSCE induced by its progressive stretching along its longest axis.^56^ This phenomenon brings both fluorophores and mesogens closer in space, favoring the intermolecular interactions between each other. In our previous carbazole-containing LSCEs exhibiting negative mechanofluorescence, the mechanical event induced quenching of the emission of the carbazole units, resulting, in turn, in the overall suppression of the fluorescence of the whole material at the macroscale.

In this case, however, both fluorophores **Cbz-sb-In-C6** and **Cbz-db-In-C6** have been designed in such a way that their fluorescence emission is not affected by the presence of the mesogens. Besides, as discussed above, the emission of **Cbz-sb-In-C6** is not influenced by the viscosity of the medium, while that of **Cbz-db-In-C6** depends noticeably on the amount of intramolecular rotation enabled by the local environment where it is located. Accordingly, the deformation of the elastomer **E-sb-1** slightly modifies the amount of light emitted by the fluorogenic units, and thus, this LSCE exhibits a weak mechanofluorescence. In contrast, the application of a uniaxial force to the nematic LSCE that integrates the FMR **Cbz-db-In-C6** places the fluorophores in a more confined microenvironment owing to the approximation of the surrounding mesogenic units. Before the stretching of this system, the fluorophores have more rotational freedom and can dissipate their excitation energy in a nonradiative fashion. Contrarily, radiative deexcitation predominates when the elastomer is stretched as the local environment becomes more restricted. Accordingly, the uniaxial deformation of LSCE **E-db-1** produces a considerable increase in the emission of the fluorophores and enhances the luminescence of the whole material. Thus, and as we hypothesized, the incorporation of FMRs into liquid crystalline elastomeric networks is a valuable and innovative strategy to program positive mechanofluorescence into LSCEs.

**Figure 8:**
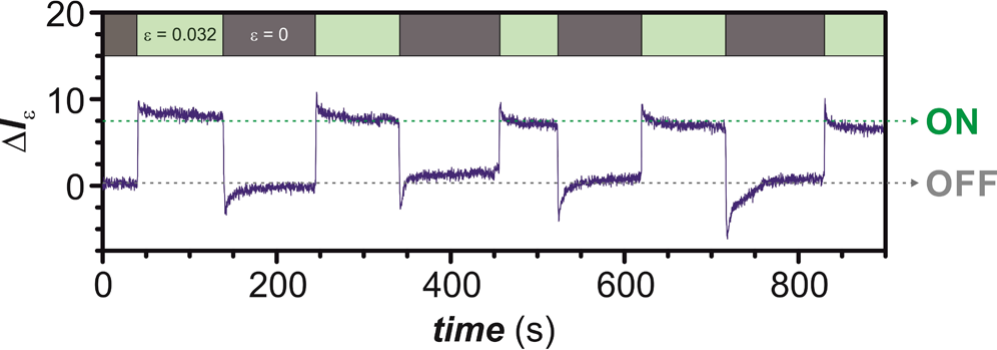
Time-resolved mechanofluorescence experiment (λ_Ex_ = 400 nm, λ_Em_ = 450 nm, applied deformation ε = 0.032) for the nematic LSCE **E-db-1** under ambient conditions.

The response time of the material was determined by means of time-resolved mechanofluorescence measurements. In these experiments, the evolution of the material fluorescence at λ_Em_ = 450 nm (λ_Ex_ = 400 nm) is monitored over time before and after the application of a specific deformation along the director direction. Figure 8 shows the time-resolved mechanofluorescence experiment for nematic LSCE **E-db-1**. This experiment has not been performed for **E-sb-1** since this elastomer does not modify its fluorescent emission upon mechanical stimulation. Experimental data evidence that fluorescence changes rapidly either when the external mechanical force is applied (ε = 0.032) or when it is released. Indeed, characteristic times of about 1 s have been estimated for both processes. In this way, these mechanoresponsive materials can complete a full operating cycle in approximately a few seconds. However, it should be mentioned that the sample requires a slightly longer time (*ca*. 20 s) to recover its initial state when the mechanical force is removed due to the slow relaxation of the sample back to the equilibrium position; this feature is not observed when the mechanical force is applied. The repeatability of the mechanofluorescence phenomenon has been tested by stretching and relaxing the LSCE in a periodic fashion over 5 cycles. Figure 8 reveals that the continuous work of the system does not affect either its intensity change upon deformation Δ*I*_ε_ or its operating time. Therefore, the design schemes presented here have evolved into a series of fluorescence-based force sensors capable of increasing their emission intensity upon the application of mechanical forces.

## Conclusions

Carbazole-indolenine dyads are valuable organic fluorophores to engineer nematic LSCEs exhibiting positive mechanofluorescence, that is, elastomeric materials that increase their fluorescent emission upon deformation. For this purpose, we have explored two fluorophores with very distinguished fluorescent characteristics as a result of the different connections of their two heterocyclic platforms. The fluorescence of the system in which the two heterocycles are directly linked through a C–C bond is barely unaffected by environmental viscosity. In contrast, the introduction of a π-conjugated bridge between the two rings makes the resulting fluorophore behave as an FMR. Accordingly, the integration of this latter fluorophore into nematic LSCEs results in a flexible luminescent material that intensifies its emission upon deformation (positive mechanofluorescence). The positive mechanofluorescence observed for this elastomer is a milestone since it is the first material of its kind. In this context, other series of FMRs will be explored and incorporated into LSCEs to get further insight into the photophysical events controlling this behavior, aiming for better mechanofluorescent materials with improved performances and unprecedented abilities.

## Supporting Information Available

Supporting Information Available: Synthesis of the monomers and fluorophores, absorption and emission spectroscopies, determination of the fluorescence quantum yield, preparation and characterization of the LSCEs, and mechanofluorescence experiments (PDF)
