# Supplementary material for: Programming Positive Mechanofluorescence in Liquid Crystalline Elastomers
Source: ACS Appl Polym Mater. 2023 Aug 2;5(8):6484–92. doi: 10.1021/acsapm.3c01050 (PMC11093412; doi:10.1021/acsapm.3c01050)
Supplement: Supplementary file 1 — ap3c01050_si_001.pdf [file ap3c01050_si_001.pdf]

Supporting Information

Programming positive mechanofluorescence in liquid crystalline elastomers

Pedro Güixens-Gallardo[a,b], Ignacio Brea[a], Jordi Manrique[a], Farhad Shohraty[a], Jaume Garcia-Amorós[a,b,]* and Dolores Velasco[a,b,]*

[a] *Grup de Materials Orgànics, Departament de Química Inorgànica i Orgànica (Secció de Química Orgànica), Universitat de Barcelona, Martí i Franquès 1, E-08028, Barcelona, Spain*

[b] *Institut de Nanociència i Nanotecnologia (IN²UB), Universitat de Barcelona, E-08028, Barcelona, Spain*

e-mail: *jgarciaamoros@ub.edu; dvelasco@ub.edu*

Materials and methods. Chemicals were purchased from commercial sources and used as received with the exception of THF, CH_2Cl_2 and DMF. THF was distilled over sodium and benzophenone under a nitrogen atmosphere. CH_2Cl_2 was distilled over CaH_2 under a nitrogen atmosphere. Commercially available anhydrous DMF was stored over activated 4 Å molecular sieves under a nitrogen atmosphere. Thiophene-free toluene was prepared by washing commercially available toluene with concentrated sulfuric acid until the acid layer was colorless. The organic layer was washed twice with water, once with a solution of potassium carbonate (10% w/w), again with water, dried over anhydrous $CaCl_2$ and finally distilled through an efficient column. Thiophene-free toluene was stored over activated 5 Å molecular sieves. All reactions were monitored by thin-layer chromatography using silica gel 60 F_{254} plates (Merck) and visualized under UV light (254 nm or 366 nm). Column chromatography was performed over silica gel (VWR, 40–63 µm). NMR spectra were recorded with a Varian Mercury 400 spectrophotometer. Chemical shifts (δ) were referred to the residual solvent signal. High-resolution mass spectra (HRMS) were recorded with a LC/MSD-TOF Agilent Technologies spectrometer by means of the electrospray ionization (ESI) technique.

Synthesis of the nematic mesogen and the isotropic cross-linker. Both the nematic mesogen 4-methoxyphenyl 4-(3-butenyloxy)benzoate and the isotropic cross-linker 1,4-di-(10-undecenyloxy)benzene (**M4OMe** and **CL** respectively in Figure S1) were prepared following already stablished protocols.[1,2]

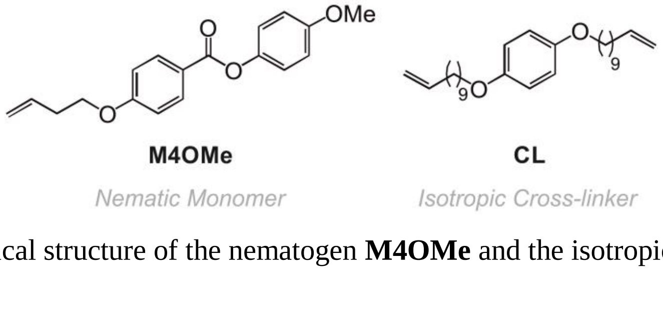

Figure S1. Chemical structure of the nematogen **M4OMe** and the isotropic cross-linker **CL**.

Synthesis of the fluorophores

***9-ethyl-3-(5-methoxy-3,3-dimethyl-3H-indol-2-yl)-9H-carbazole* (Cbz-sb-In-OMe).** A solution of 1-(9-ethyl-9*H*-carbazol-3-yl)-2-methylpropan-1-one (2.307 g, 8.69 mmol), 4-methoxyphenylhydrazine hydrochloride (1.518 g, 8.69 mmol) and *p*-toluenesulfonic acid monohydrate (11.8 mg, 0.062 mmol) in absolute ethanol (80 mL) was heated under reflux for 21 h. After cooling down to ambient temperature, the solvent was evaporated under reduced pressure. The crude was diluted with a saturated aqueous solution of $NaHCO_3$ and the product was extracted with dichloromethane. The combined organic extract was dried over anhydrous Na_2SO_4, filtered and the solvent was evaporated under reduced pressure. The crude was purified by flash column chromatography using a mixture of dichloromethane and hexane (7:3 v/v) as eluent to afford **Cbz-sb-In-OMe** (59%, 1.902 g, 5.16 mmol). ^{1}H NMR (400 MHz, $CDCl_3$): δ 8.89 (d, J = 2 Hz, 1 H, Ar *H*), 8.31 (dd, J = 9 Hz, 2 Hz, 1 H, Ar *H*), 8.20 (d, J = 8 Hz, 1 H, Ar *H*), 7.61 (d, J = 8 Hz, 1 H, Ar *H*), 7.43-7.53 (m, 3 H, Ar *H*), 7.26-7.31 (m, 1 H, Ar *H*), 6.89-6.93 (m, 2 H, Ar *H*), 4.42 (q, J = 7 Hz, 2 H, CH_2), 3.88 (s, 3 H, OCH_3), 1.70 (s, 6 H, $(CH_3)_2C$), 1.48 (t, J = 7 Hz, 3 H, CH_3) ppm. HRMS (ESI) *m/z* for $C_{25}H_{25}N_2O$ $[M+H]^+$; calcd.: 369.1967; found: 369.1963.

***2-(9-ethyl-9H-carbazol-3-yl)-3,3-dimethyl-3H-indol-5-ol* (Cbz-sb-In-OH). Cbz-sb-In-OMe** (1.000 g, 2.71 mmol) was dissolved in anhydrous CH_2Cl_2 (40 mL) under nitrogen atmosphere at 0 ºC. After, a solution of BBr_3 (1 M in CH_2Cl_2, 4.07 mL, 4.07 mmol) was added dropwise. Then, the mixture was stirred at 0 ºC for 1 h and at room temperature for 14 h. The reaction was quenched by adding a saturated aqueous solution of $NaHCO_3$ and the product was extracted with CH_2Cl_2. The combined organic extract was dried over anhydrous Na_2SO_4, filtered and the solvent was evaporated under reduced pressure. The crude was purified by flash column chromatography using a mixture of

CH_2Cl_2 and ethyl acetate (97:3 v/v) as eluent to afford **Cbz-sb-In-OH** (69%, 0.661 g, 1.86 mmol). ^{1}H NMR (400 MHz; $CDCl_3$): δ 8.88 (d, *J* = 2 Hz, 1 H, Ar *H*), 8.30 (dd, *J* = 9 Hz, 2 Hz, 1 H, Ar *H*), 8.19 (d, *J* = 8 Hz, 1 H, Ar *H*), 7.55 (d, *J* = 8 Hz, 1 H, Ar *H*), 7.43-7.53 (m, 3 H, Ar *H*), 7.26-7.31 (m, 1 H, Ar *H*), 6.87 (d, *J* = 2 Hz, 1 H, Ar *H*), 6.81 (dd, *J* = 8 Hz, 2 Hz, 1 H, Ar *H*), 4.89 (s, 1 H, O*H*), 4.41 (q, *J* = 7 Hz, 2 H, CH_2), 1.69 (s, 6 H, $(CH_3)_2C$), 1.48 (t, *J* = 7 Hz, 3 H, CH_3) ppm. HRMS (ESI) *m*/*z* for $C_{24}H_{23}N_2O$ $[M+H]^+$; calcd.: 355.1810; found: 355.1805.

***9-ethyl-3-(5-(hex-5-en-1-yloxy)-3,3-dimethyl-3H-indol-2-yl)-9H-carbazole* (Cbz-sb-In-C6). Cbz-sb-In-OH** (200 mg, 0.56 mmol) and K_2CO_3 (156 mg, 1.13 mmol) were mixed in anhydrous DMF (6 mL) under nitrogen atmosphere and stirred at room temperature for 30 minutes. Then, 6-bromo-1-hexene (0.19 mL, 1.42 mmol) was added and the reaction mixture was stirred at room temperature overnight. The reaction was diluted with water and the product was extracted with ethyl acetate. The combined organic extract was dried over anhydrous Na_2SO_4, filtered and the solvent was evaporated under reduced pressure. The crude was purified by flash column chromatography using a mixture of CH_2Cl_2 and ethyl acetate (9:1 v/v) as eluent to afford **Cbz-sb-In-C6** (88%, 216 mg, 0.49 mmol). ^{1}H NMR (400 MHz; $CDCl_3$): δ 8.89 (s, 1 H, Ar *H*), 8.31 (d, *J* = 9 Hz, 1 H, Ar *H*), 8.20 (d, *J* = 8 Hz, 1 H, Ar *H*), 7.60 (d, *J* = 8 Hz, 1 H, Ar *H*), 7.43-7.53 (m, 3 H, Ar *H*), 7.26-7.29 (m, 1 H, Ar *H*), 6.93 (d, *J* = 2 Hz, 1 H, Ar *H*), 6.89 (dd, *J* = 8 Hz, 2 Hz, 1 H, Ar *H*), 5.81-5.91 (1 H, m, =C*H*), 4.98-5.08 (m, 2 H, $=CH_2$), 4.41 (q, *J* = 7 Hz, 2 H, NCH_2), 4.03 (t, *J* = 6 Hz, 2 H, OCH_2), 2.14-2.19 (m, 2 H, CH_2), 1.81-1.88 (m, 2 H, CH_2), 1.69 (s, 6 H, $(CH_3)_2C$), 1.58-1.66 (m, 2 H, CH_2), 1.48 (t, *J* = 7 Hz, 3 H, CH_3) ppm. HRMS (ESI) *m*/*z* for $C_{30}H_{33}N_2O$ $[M+H]^+$; calcd.: 437.2593; found: 437.2590.

***9-ethyl-3-(2-(5-methoxy-3,3-dimethyl-3H-indol-2-yl)vinyl)-9H-carbazole* (Cbz-db-In-OMe).** A solution of 5-methoxy-2,3,3-trimethyl-3*H*-indole (2.000 g, 10.6 mmol), 9-

ethyl-9*H*-carbazole-3-carbaldehyde (2.300 g, 10.3 mmol) and trifluoroacetic acid (0.9 mL, 11.8 mmol) in absolute ethanol (30 mL) was stirred under reflux for 24 hours. After cooling down to ambient temperature, the solvent was evaporated under reduced pressure. The crude was diluted with a saturated aqueous solution of $NaHCO_3$ and the product was extracted with dichloromethane. The combined organic extract was dried over anhydrous Na_2SO_4, filtered and the solvent was evaporated under reduced pressure. The crude was purified by flash column chromatography using a mixture of hexane and ethyl acetate (8:2 v/v) as eluent to afford **Cbz-db-In-OMe** (48%, 1.969 g, 4.99 mmol). ^{1}H NMR (400 MHz; acetone-d_6): δ 8.55 (d, J = 2 Hz, 1 H, Ar *H*), 8.22 (d, J = 8 Hz, 1 H, Ar *H*), 7.92 (d, J = 16 Hz, 1 H, =C*H*), 7.88 (dd, J = 9 Hz, 2 Hz, 1 H, Ar *H*), 7.62 (d, J = 9 Hz, 1 H, Ar *H*), 7.60 (d, J = 9 Hz, 1 H, Ar *H*), 7.49 (ddd, J = 8 Hz, 8 Hz, 1 Hz, 1 H, Ar *H*), 7.43 (d, J = 8 Hz, 1 H, Ar *H*), 7.25 (d, J = 16 Hz, 1 H, =C*H*), 7.23-7.29 (m, 2 H, Ar *H*), 7.05 (d, J = 2 Hz, 1 H, Ar *H*), 6.88 (dd, J = 8 Hz, 2 Hz, 1 H, Ar *H*), 4.52 (q, J = 7 Hz, 2 H, CH_2), 3.84 (s, 3 H, OCH_3), 1.49 (s, 6 H, (CH_3)$_2$C), 1.43 (t, J = 7 Hz, 3 H, CH_3) ppm. HRMS (ESI) *m*/*z* for $C_{27}H_{27}N_2O$ $[M+H]^+$; calcd.: 395.2123; found: 395.2114.

2-(2-(9-ethyl-9H-carbazol-3-yl)vinyl)-3,3-dimethyl-3H-indol-5-ol **(Cbz-db-In-OH).** Sodium hydride (1.100 g, 27.5 mmol, 60% dispersion in mineral oil) was suspended in anhydrous DMF (65 mL) at 0 ºC under nitrogen atmosphere. Ethanethiol (2.0 mL, 27.7 mmol) was added dropwise and left stirring at this temperature until the evolution of hydrogen ceased. Then, a solution of **Cbz-db-In-OMe** (2.300 g, 5.83 mmol) in anhydrous DMF (17.5 mL) was added to the sodium ethanothiolate solution. The resulting mixture was stirred at 125 ºC for 3 hours. After cooling down to ambient temperature, the reaction was diluted with water and acidified until pH = 2 with an aqueous solution of HCl (2 M). The product was extracted with dichloromethane. The combined organic extract was dried over anhydrous Na_2SO_4, filtered and the solvent was

evaporated. The crude was purified by flash column chromatography using a mixture of hexane and ethyl acetate (7:3 v/v) as eluent to afford **Cbz-db-In-OH** (74%, 1.650 g, 4.34 mmol). ^{1}H NMR (400 MHz; acetone-d_6): δ 8.53 (d, *J* = 2 Hz, 1 H, Ar *H*), 8.42 (s, 1 H, O*H*), 8.21 (d, *J* = 8 Hz, 1 H, Ar *H*), 7.89 (d, *J* = 16 Hz, 1 H, =C*H*), 7.87 (dd, *J* = 9 Hz, 2 Hz, 1 H, Ar *H*), 7.62 (d, *J* = 9 Hz, 1 H, Ar *H*), 7.59 (d, *J* = 9 Hz, 1 H, Ar *H*), 7.49 (ddd, *J* = 8 Hz, 8 Hz, 1 Hz, 1 H, Ar *H*), 7.35 (d, *J* = 8 Hz, 1 H, Ar *H*), 7.25 (ddd, *J* = 8 Hz, 8 Hz, 1 Hz, 1 H, Ar *H*), 7.23 (d, *J* = 16 Hz, 1 H, =C*H*), 6.91 (d, *J* = 2 Hz, 1 H, Ar *H*), 6.79 (dd, *J* = 8 Hz, 2 Hz, 1 H, Ar *H*), 4.51 (q, *J* = 7 Hz, 2 H, CH_2), 1.47 (s, 6 H, $(CH_3)_2C$), 1.43 (t, *J* = 7 Hz, 3 H, CH_3) ppm. HRMS (ESI) *m*/*z* for $C_{26}H_{25}N_2O$ $[M+H]^+$; calcd.: 381.1967; found: 381.1970.

***9-ethyl-3-(2-(5-(hex-5-en-1-yloxy)-3,3-dimethyl-3H-indol-2-yl)vinyl)-9H-carbazole* (Cbz-db-In-C6). Cbz-db-In-OH** (500 mg, 1.31 mmol), K_2CO_3 (193 mg, 1.40 mmol) were mixed in anhydrous DMF (10 mL) under nitrogen atmosphere and stirred at room temperature for 30 minutes. Then, 6-bromo-1-hexene (0.20 mL, 1.50 mmol) was added and the reaction mixture was stirred at 80 ºC overnight. After cooling down to ambient temperature, the reaction was diluted with water and the product was extracted with ethyl acetate. The combined organic extract was dried over anhydrous Na_2SO_4, filtered and the solvent was evaporated under reduced pressure. The crude was purified by flash column chromatography using a mixture of hexane and ethyl acetate (9:1 v/v) as eluent to afford **Cbz-db-In-C6** (81%, 492 mg, 1.06 mmol). ^{1}H NMR (400 MHz; $CDCl_3$): δ 8.31 (d, *J* = 1 Hz, 1 H, Ar *H*), 8.13 (d, *J* = 8 Hz, 1 H, Ar *H*), 7.85 (d, *J* = 16 Hz, 1 H, =C*H*), 7.75 (dd, *J* = 9 Hz, 1 Hz, 1 H, Ar *H*), 7.52 (d, *J* = 8 Hz, 1 H, Ar *H*), 7.40-7.51 (m, 3 H, Ar *H*), 7.26-7.29 (m, 1 H, Ar *H*), 7.10 (d, *J* = 16 Hz, 1 H, =C*H*), 6.88 (d, *J* = 2 Hz, 1 H, Ar *H*), 6.85 (dd, *J* = 8 Hz, 2 Hz, 1 H, Ar *H*), 5.80-5.90 (1 H, m, =C*H*), 4.96-5.08 (m, 2 H, =CH_2), 4.39 (q, *J* = 7 Hz, 2 H, NCH_2), 4.01 (t, *J* = 6 Hz, 2 H, OCH_2), 2.13-2.19 (m, 2 H, CH_2), 1.80-

1.87 (m, 2 H, CH_2), 1.58-1.66 (m, 2 H, CH_2), 1.50 (s, 6 H, (CH_3)$_2$C), 1.46 (t, J = 7 Hz, 3 H, CH_3) ppm. HRMS (ESI) m/z for $C_{32}H_{35}N_2O$ $[M+H]^+$; calcd.: 463.2749; found: 46.2752.

Absorption and emission spectroscopies. Absorption spectra were recorded with a Varian Cary 500 E UV-Vis-NIR spectrophotometer. Emission spectra were recorded with a PTI 810 Series spectrophotometer (Birmingham, USA) controlled by a PC equipped with the PTI Felix32 software, under aerated conditions. All spectroscopic measurements were performed in 1 cm optical path quartz cuvettes (Hellma Analytics).

Determination of the fluorescence quantum yields (Φ_f) was performed using quinine sulfate in 0.1 M $HClO_4$ (Φ_f = 0.60 at 20-40 ºC) as a standard.[3] The excitation wavelength was 365 nm and the emission spectra were recorded in the range of 380 - 600 nm. The absorption of the distinct solutions was kept below 0.10 to prevent inner filter effects. The quantum yields were calculated using equation (1): [4]

$$\Phi_{f,x} = \Phi_{f,st} \frac{F_x}{F_{st}} \frac{1-10^{-Abs_{st}}}{1-10^{-Abs_x}} \frac{n_x^2}{n_{st}^2} \qquad (1)$$

where Φ_f is the quantum yield, F is the integrated fluorescence intensity, Abs is the absorbance of solution at the excitation wavelength, n is the refractive index of the solvent. The subscripts x and st stand for the sample and standard, respectively. The measurements were triplicated.

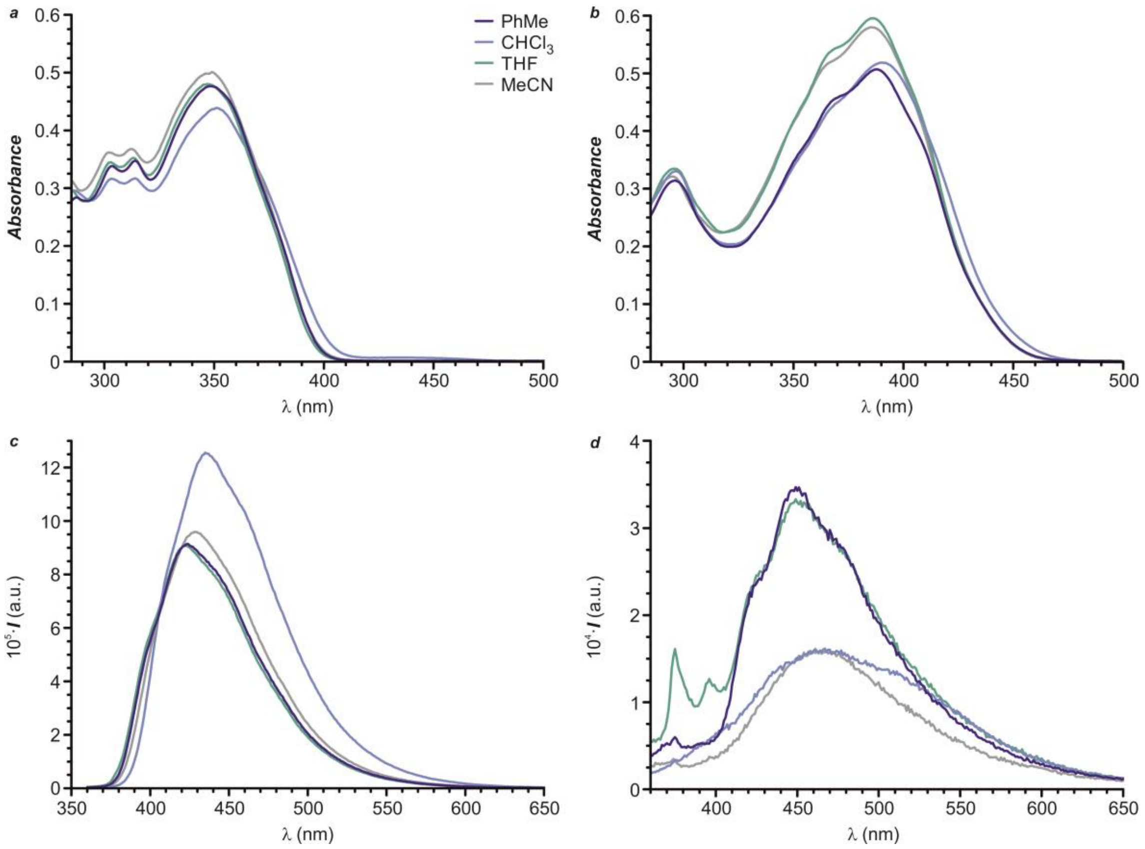

Figure S2. Absorption (***a*** and ***b***) and emission (***c*** and ***d***, λ_{Ex} = 340 nm) spectra of 20 µM solutions of **Cbz-sb-In-OMe** (***a*** and ***c***) and **Cbz-db-In-OMe** (***b*** and ***d***) in different solvents.

Preparation of the LSCEs. A solution of the distinct monomers, i.e. **M4OMe** (89% mol), **CL** (10% mol) and the corresponding fluorophore (**Cbz-sb-In-C6** or **Cbz-db-In-C6**, 1% mol), and polyhydrogenomethylsiloxane (~85 Si-H groups per chain, AB101031, purchased from abcr) in thiophene-free toluene (1 mL) was placed in a Teflon mould. A solution of cyclooctadieneplatinum (II) chloride in CH_2Cl_2 (1% w/w, 40 µL) was added and the reaction mixture was heated in an oven at 75 ºC for 30 minutes. Then, the mold was cooled down to room temperature and the elastomer (not totally cross-linked) was carefully removed from the mold. During the deswelling process, a uniaxial force was applied to the hung elastomer, parallel to its longest axis, in order to achieve a macroscopic orientation of the nematic directors. After, the cross-linking reaction was completed by leaving the elastomer under load in an oven at 75 ºC for 2 days. The non-

reacted monomers were removed from the network by a swelling-deswelling process using toluene and hexane, respectively.

Characterization of the LSCEs. DSC thermograms were recorded with a Mettler-Toledo DSC821 calorimeter at a scan rate of 10 ºC·min^{-1} under a nitrogen flow. Polarized optical microscopy (POM) was carried out at room temperature using a Nikon Eclipse polarizing microscope. POM experiments were run by rotating the analyzer of the microscope with respect to the longest axis of the elastomeric sample. X-ray scattering experiments were performed in a PANalytical X'Pert PRO MPD θ/θ powder diffractometer (radius = 240 mm) with a PIXcel detector (active length = 3.347º) in a convergent beam configuration and a transmission geometry. All LSCEs were sandwiched between low absorbing polyester films (thickness = 3.6 µm). X-Ray scattering patterns were registered at room temperature with the monochromatic Cu Kα radiation (λ = 1.5418 Å) at an operating power of 45 kV (40 mA). Slits were adjusted in such a way that the height of the resulting incident beam was equal to 400 µm. A mask to define a beam length, in the axial direction, of about 4 mm was also used. 2θ/θ scans were registered from 2θ = 1º to 2θ = 60 º with a step size of 2θ = 0.026º and a measuring time of 300 s per step. On the other hand, all azimuthal scans were collected at a step size of φ = 1º and a measuring time of 2.55 s per step. From the azimuthal distribution of intensities at a scattering vector corresponding to the maximum of the reflex located at 2θ = 19.7−20.0º, which follows a Gaussian function, the angular distribution of the mesogens with respect to the director can be calculated. The order parameter, *S*, has been determined using the method reported by Lovell and Mitchell.[5,6] In the swelling experiments, the dimensions of the network in the deswollen and swollen state in toluene were measured at room temperature with a graduated magnifying glass.

XRD diffraction

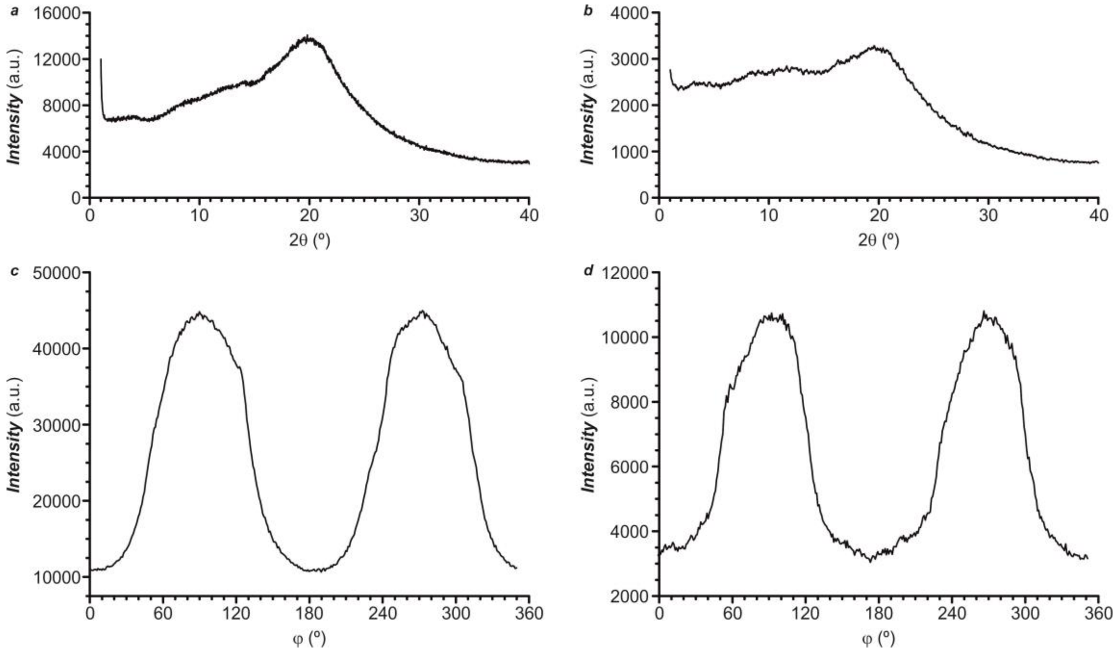

Figure S3. X-ray diffraction pattern (***a*** and ***b***) and azimuthal intensity distribution of the wide angle reflex at 2θ = 19.7º (***c*** and ***d***, spacing = 4.5 Å) for the liquid single crystal elastomer **E-sb-1** (***a*** and ***c***) and **E-db-1** (***b*** and ***d***).

Polarized optical microscopy

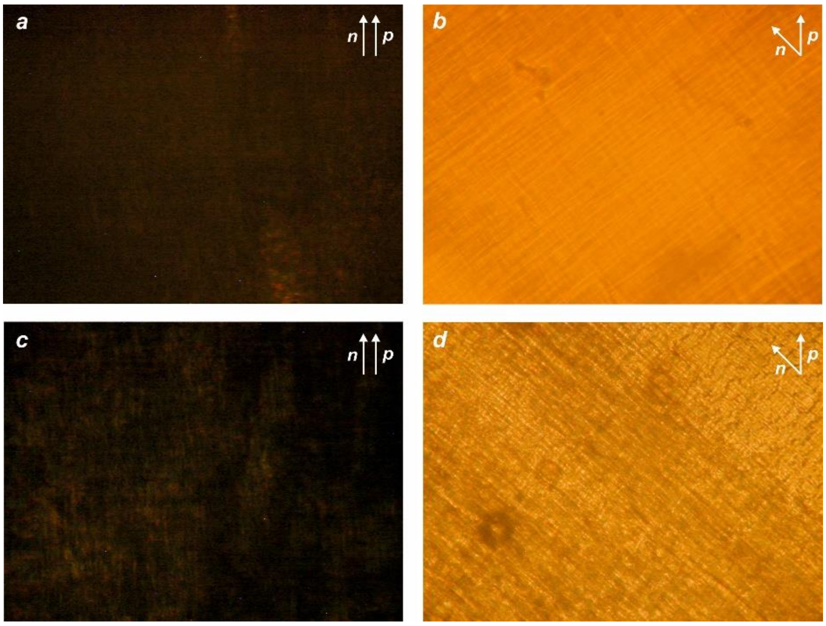

Figure S4. Photomicrographs of **E-sb-1** (***a*** and ***b***) and **E-db-1** (***c*** and ***d***) at different angles between the polarization vector, ***p***, of the analyzer and the nematic director, ***n***.

Differential scanning calorimetry

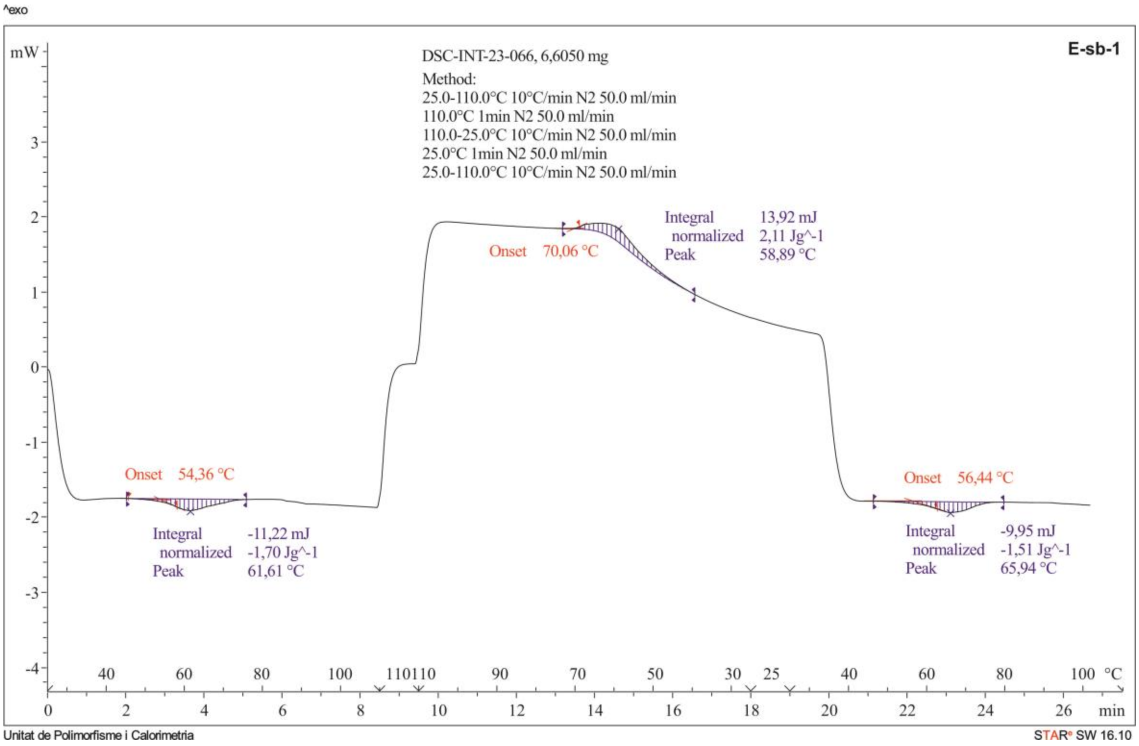

Figure S5. DSC thermogram for LSCE **E-sb-1** registered at 10 ºC·min^{-1}.

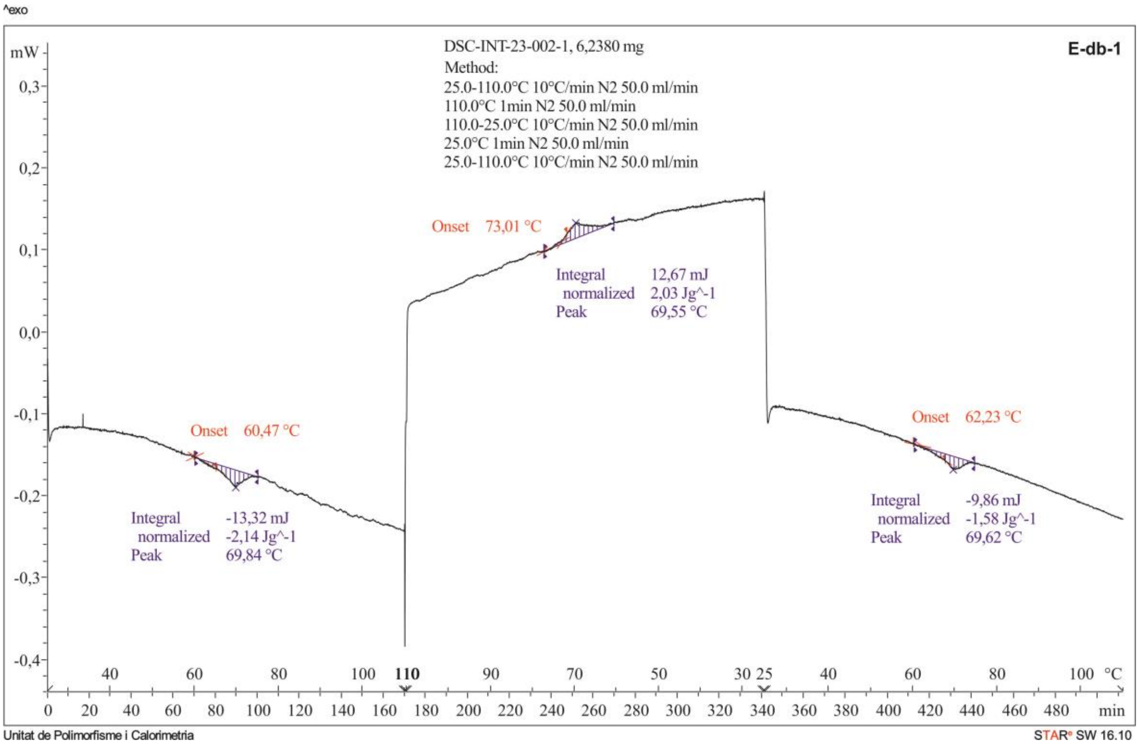

Figure S6. DSC thermogram for LSCE **E-db-1** registered at 10 ºC·min^{-1}.

Mechanofluorescence of the LSCEs. Mechanofluorescence experiments were performed in a PTI 810 Series spectrophotometer (see above) controlled by a PC equipped with the PTI Felix32 software. All mechanofluorescent experiments were carried out by gluing the elastomeric samples by both ends into a self-constructed setup. LSCE samples were attached to the sample holder the previous day of the experiment to ensure thermodynamic equilibrium in the system. The sample was placed inside the spectro-photometer in a front-face geometry. In all instances, the fluorophores were excited at λ_{Ex} = 365 nm; the resulting luminescence was collected from 400 to 600 nm. All experiments were carried out under ambient conditions. Once the first emission spectrum was collected a uniaxial deformation along the director direction of the LSCE was applied stepwise and the variation in the emission intensity was monitored from 400 to 600 nm. After deformation, the elastomeric sample was left to equilibrate for a minimum of 15 minutes prior to each spectrum collection. The collected spectra were later transferred to a commercially available software (Origin version 2018) for further data treatment.

References

[1] Gray, G. W.; Jones, B. The mesomorphic transition points of the *para*-normal-alkoxybenzoic acids - a correction. *J. Chem. Soc.* **1953**, 4179−4180.

[2] Squires, A. M.; Tajbakhsh, A. R.; Terentjev, E. M. Dynamic shear modulus of isotropic elastomers. *Macromolecules* **2004**, *37*, 1652−1659.

[3] Nawara, K.; Waluk, J. Goodbye to quinine in sulfuric acid solutions as a fluorescence quantum yield standard. *Anal. Chem.* **2019,** *91*, 5389−5394.

[4] Würth, C.; Grabolle, M.; Pauli, J.; Spieles, M.; Resch-Genger, U. Relative and absolute determination of fluorescence quantum yields of transparent samples. *Nat. Protoc.* **2013**, *8*, 1535−1550.

[5] Lovell, R.; Mitchell, G. R. Molecular orientation distribution derived from an arbitrary reflection. *Acta Cryst.* **1981**, *A37*, 135−137.

[6] Mitchell, G. R.; Windle, A. *Orientation in Liquid Crystal Polymers*, in *Developments in Crystal Polymers* – 2, Basset, D. C. (Ed.), Science, London, New York, **1988**.
